# Promoting Activity, Independence, and Stability in Early Dementia and mild cognitive impairment (PrAISED): randomised controlled trial

**DOI:** 10.1136/bmj-2023-074787

**Published:** 2023-08-29

**Authors:** Rowan H Harwood, Sarah E Goldberg, Andrew Brand, Veronika van Der Wardt, Vicky Booth, Claudio Di Lorito, Zoe Hoare, Jennie Hancox, Rupinder Bajwa, Clare Burgon, Louise Howe, Alison Cowley, Trevor Bramley, Annabelle Long, Juliette Lock, Rachael Tucker, Emma J Adams, Rebecca O’Brien, Fiona Kearney, Katarzyna Kowalewska, Maureen Godfrey, Marianne Dunlop, Kehinde Junaid, Simon Thacker, Carol Duff, Tomas Welsh, Annette Haddon-Silver, John Gladman, Pip Logan, Kristian Pollock, Kavita Vedhara, Victoria Hood, Roshan Das Nair, Helen Smith, Rhiannon Tudor-Edwards, Ned Hartfiel, Victory Ezeofor, Robert Vickers, Martin Orrell, Tahir Masud

**Affiliations:** 1 School of Health Sciences, University of Nottingham, Queen’s Medical Centre, Nottingham, NG7 2HA, UK; 2 Nottingham University Hospitals NHS Trust, Queen’s Medical Centre, Nottingham, UK; 3 North Wales Organisation for Randomised Trials in Health, Bangor University, Bangor, Gwynedd, UK; 4 Department of General, Preventative and Rehabilitation Medicine, Philipps-Universität Marburg 35032 Marburg, Germany; 5 School of Medicine, University of Nottingham, Queen’s Medical Centre, Nottingham, UK; 6 School of Sport, Exercise and Health Sciences, Loughborough University, Loughborough, UK; 7 Nottinghamshire Healthcare NHS Foundation Trust, Lings Bar Hospital, Gamston, Nottingham, UK; 8 Public representative, Nottingham, UK; 9 Mental Health Services for Older People, Nottinghamshire Healthcare NHS Foundation Trust, Highbury Hospital, Nottingham, UK; 10 Centre for Research and Development, Derbyshire Healthcare NHS Foundation Trust, Kingsway Hospital, Derby, UK; 11 Lincolnshire Partnership NHS Foundation Trust, Lincoln, UK; 12 The RICE Centre, Research Institute for the Care of Older People, Royal United Hospital, Bath, UK; 13 Oxford Health NHS Foundation Trust, Research and Development, Warneford Hospital, Oxford, UK; 14 Centre for Academic Primary Care, Lifespan and Population Health, University of Nottingham, Nottingham, UK; 15 SINTEF, Torgarden, Trondheim, Norway; 16 Centre for Health Economics and Medicines Evaluation, College of Health and Behavioural Sciences, Bangor University, Bangor, Gwynedd, UK; 17 Institute for Mental Health, University of Nottingham, Nottingham, UK

## Abstract

**Objective:**

To determine the effectiveness of an exercise and functional activity therapy intervention in adults with early dementia or mild cognitive impairment compared with usual care.

**Design:**

Randomised controlled trial.

**Setting:**

Participants’ homes and communities at five sites in the United Kingdom.

**Participants:**

365 adults with early dementia or mild cognitive impairment who were living at home, and family members or carers.

**Intervention:**

The intervention, Promoting activity, Independence, and Stability in Early Dementia and mild cognitive impairment (PrAISED), was a specially designed, dementia specific, rehabilitation programme focusing on strength, balance, physical activity, and performance of activities of daily living, which was tailored and progressive and addressed risk and the psychological needs of people with dementia. Up to 50 therapy sessions were provided over 12 months. The control group received usual care plus a falls risk assessment. Procedures were adapted during the covid-19 pandemic.

**Main outcome measures:**

The primary outcome was score on the carer (informant) reported disability assessment for dementia scale 12 months after randomisation. Secondary outcomes were self-reported activities of daily living, physical activity, quality of life, balance, functional mobility, fear of falling, frailty, cognition, mood, carer strain, service use at 12 months, and falls between months 4 and 15.

**Results:**

365 patient participants were randomised, 183 to intervention and 182 to control. The median age of participants was 80 years (range 65-95), median Montreal cognitive assessment score was 20 out of 30 (range 13-26), and 58% (n=210) were men. Intervention participants received a median of 31 therapy sessions (interquartile range 22-40) and reported completing a mean 121 minutes of PrAISED exercise each week. Primary outcome data were available for 149 intervention and 141 control participants. Scores on the disability assessment for dementia scale did not differ between groups: adjusted mean difference −1.3, 95% confidence interval −5.2 to 2.6; Cohen’s d effect size −0.06, 95% confidence interval −0.26 to 0.15; P=0.51). Upper 95% confidence intervals excluded small to moderate effects on any of the range of outcome measures. Between months 4 and 15 the intervention group experienced 79 falls and the control group 200 falls (adjusted incidence rate ratio 0.78, 95% confidence interval 0.5 to 1.3; P=0.3).

**Conclusion:**

The intensive PrAISED programme of exercise and functional activity training did not improve activities of daily living, physical activity, or quality of life; reduce falls; or improve any other secondary health status outcomes, despite good uptake. Future research should consider alternative approaches to maintaining ability and wellbeing in people with dementia.

**Trial registration:**

ISRCTN Registry ISRCTN15320670.

## Introduction

Dementia results in progressive loss of ability in activities of daily living and physical activity. Multiple mechanisms lead to functional loss, including cognitive and neurological decline, comorbidities, acute illness, injuries, delirium, inactivity, deconditioning, and restriction of opportunities, especially if people experience stigma or family members are concerned about safety. Dementia (and a possible precursor state, mild cognitive impairment) confers an increased risk of crises, including acute physical illness and a twofold increased risk of falls.^1^ ^2^ ^3^ ^4^ ^5^ Each year, 60-80% of people with dementia fall.^1^ ^2^ ^3^ ^4^ ^5^

The prevalence of dementia increases exponentially with age, with 20% of 80 year olds affected.^6^ ^7^ A similar proportion have mild cognitive impairment.^6^ ^7^ Prevalence is expected to double in the next 30 years.^8^ Dementia is one of the main drivers of dependency—the need for help from other people. It creates high levels of demand on health and social care services, families, and other informal carers.^9^ The English National Dementia Strategy emphasised the importance of early diagnosis and the goal of living well with dementia.^10^ Reasons to diagnose dementia include timely access to cognitive enhancing drugs and cognitive stimulation therapy, but the effects of these are small.^11^ ^12^ Commentators have highlighted a relative lack of available therapeutic interventions.^13^ Exercise has been proposed as a way of preventing or slowing the progression of dementia^14^ but has been shown to have little effect on global cognition in trials.^15^ ^16^ ^17^ ^18^ Some evidence suggests that it might slow the decline in performance of activities of daily living or prevent falls.^15^ ^18^ ^19^

In this study we hypothesised that exercise based, functionally directed rehabilitation would improve physical reserve, promote safe performance of activities, reduce falls, and enhance recovery from intercurrent illness or injury in people with early dementia or mild cognitive impairment, and hence improve activities of daily living. To develop and evaluate this approach, we undertook a programme of research.^20^ We developed a dementia specific therapy intervention called Promoting Activity, Independence, and Stability in Early Dementia and mild cognitive impairment (PrAISED).^21^ The target population was people with relatively mild impairment who retained the capacity to participate, learn, change behaviour, and develop new routines, and thereby might benefit from the programme. We previously conducted a three arm feasibility trial with 60 participants in which we evaluated the PrAISED intervention delivered with supervision over 12 months versus a reduced schedule of three months’ supervision and a control condition. We found that intervention delivery and research were feasible and there were benefits in balance and mobility outcomes, but that to sustain adherence most participants required ongoing supervision.^21^ ^22^ ^23^ In the current randomised controlled trial we determined the effectiveness of the PrAISED intervention on, among other outcomes, activities of daily living, falls, physical activity, and quality of life.

## Methods

### Study design, setting, and participants

We performed a multicentre, individually randomised (1:1), stratified, pragmatic, parallel group, randomised controlled trial.^24^ Recruitment was paused between March and September 2020 owing to the covid-19 pandemic, and adaptations to delivery were instituted.

Trained researchers from five sites in England recruited participants through secondary care memory clinics (dementia diagnostic services), general practice registers, dementia support groups, and the National Institute for Health and Care Research Join Dementia Research register. The intervention was delivered in participants’ homes and in local communities.

Patient participants were older than 65 years, had a diagnosis of early dementia or mild cognitive impairment, were willing to undertake an exercise programme, and had a family member or carer who knew the participant and who provided a minimum of one hour of weekly contact in person or by telephone or internet and was willing to act as informant. The diagnosis of dementia or mild cognitive impairment was based on Diagnostic and Statistical Manual DSM-5 criteria, including brain imaging, in memory clinics. The neuropsychological tests that we report were done for the purposes of the trial and were not used in diagnosis. We operationalised mild severity as a Montreal cognitive assessment score of 13-25. Participants had to be able to walk without human help; be able to communicate in English; have adequate sight, hearing, and dexterity to complete neuropsychological tests; have mental capacity to give consent, as assessed by a study researcher; and consented to participate. Carers participated in their own right, and their consent was obtained separately.

Exclusion criteria included a diagnosis of dementia with Lewy bodies, a comorbidity preventing participation (such as severe breathlessness, pain, or severe neurological disorder), anticipated unavailability over the next year (eg, relocation, prolonged holiday), or life expectancy less than a year.

### Baseline data

The study dataset included multiple health status measures, as is appropriate for a complex intervention trial.^25^ ^26^ ^27^ ^28^ ^29^ ^30^ The rationale was to measure a range of credible predictor, mediator, intermediate, and distal health status outcomes, including activities of daily living, falls, balance, mobility, frailty, executive function, mood, carer strain, and quality of life.

Baseline data comprised personal characteristics, medications, medical and falls history, activities of daily living (disability assessment for dementia scale^31^ and Nottingham extended activities of daily living scale^32^), cognition (Montreal cognitive assessment scale (MoCA)^33^), animal naming verbal fluency, Cambridge neuropsychological test automated battery (CANTAB),^34^ mood (hospital anxiety and depression scale^35^), apathy evaluation scale,^36^ physical activity (Longitudinal Ageing Study of Amsterdam Physical Activity Questionnaire^37^ ^38^), step count (Misfit Shine accelerometer), self-assessed and proxy assessed quality of life (DEMQOL,^39^ DEMQOL-U,^40^ and EuroqoL EQ-5D-3L^41^), fear of falling (short falls efficacy scale-international^42^), frailty (SHARE (Survey of Health, Ageing, and Retirement in Europe) frailty instrument^43^), balance (Berg balance scale^44^), mobility and ability in divided attention (timed up and go, dual task timed up and go^45^), hand grip strength (Camry EH101 Electronic Hand Dynamometer), health and social care resource use for patient and carer (client service receipt inventory^46^), carer strain (care giver strain index^47^), and carer health related quality of life (EQ-5D-5L). Verbal fluency, apathy, and Cambridge neuropsychological test automated battery measures were intended as markers of executive function, which is associated with risk of falls and has been reported to improve with exercise interventions.^48^ ^49^ ^50^ ^51^

### Intervention

The intervention was delivered by National Health Service and other local healthcare providers, according to a manual^52^ (TIDieR checklist, see supplementary appendix 1). Centrally based research therapists trained the clinicians, which included a two day initial course, a mid-point refresher conference, and weekly teleconferences to discuss problems, share solutions, and reinforce the core principles of PrAISED.

The development and content of the PrAISED intervention have been published.^21^ ^52^ ^53^ Participants in the intervention arm received an individually tailored programme comprising physical exercises (ie, progressive strength, balance, and dual task training^54^ ^55^ ^56^ ^57^ ^58^), functional activities (ie, activities of daily living with an element of physical activity, such as going out shopping),^59^ ^60^ inclusion in community life (eg, through signposting exercise classes and facilities), risk enablement (positive risk taking),^61^ and environmental assessment (accessibility and safety problems at home). Participants received up to 50 home therapy sessions over 12 months from a multidisciplinary team comprising physiotherapists, occupational therapists, and rehabilitation support workers (assistant practitioners). Sessions were intended to teach and supervise exercise and functional activities, monitor progress, and adjust and progress the programme. Delivery used a specifically developed behaviour change model.^23^ ^62^ ^63^ ^64^ ^65^ ^66^ ^67^ The intervention was tailored to individual abilities, comorbidities, interests, and goals using a stratification tool to determine the frequency of intervention to enable participants to sustain the programme.^52^ Participants were encouraged to perform their programme for a minimum of three hours each week based on previous research findings for improvements in falls and executive function.^48^ ^49^ ^50^ ^68^ ^69^ Family members or carers were encouraged to support or participate when possible. The amount of supervision was tapered (ie, became progressively less frequent over time) to encourage habit formation and promotion of self-directed exercise and activity between supervised sessions and after the programme had finished. Visits lasted about an hour and comprised two therapist visits weekly for three months, one visit weekly for three months, one visit fortnightly for three months, and one visit monthly for three months. The intervention changed as the programme progressed. Therapy sessions were intended to be delivered in-person. Participants were encouraged to access community activities and facilities such as exercise groups, gym, or swimming as a way of maintaining engagement with exercise.

The control intervention consisted of falls prevention assessment and advice modelled on usual falls prevention care, which comprised an initial therapy visit for assessment and up to two further visits by a study therapist to review actions, give advice, and refer on to the general practitioner or local services if assessed to be clinically necessary. These visits lasted a maximum of 90 minutes. The control participants were seen by the same therapists who delivered the active intervention.

Both study groups were assessed using the Guide to Action falls risk decision support tool.^70^ Advice was offered based on the findings, including further assessment both clinically and for equipment, and medication review by the participant’s GP, if necessary. Non-study interventions were permitted in both study arms, including cognitive stimulation therapy, use of acetylcholine inhibitor or memantine drugs, and referrals to mental health, medical, rehabilitation, or falls prevention services.

### Outcome evaluation

Each participant took part for 15 months. A brief postal follow-up, with telephone support if needed, was undertaken with a carer after six months. The main follow-up was completed at 12 months (within four weeks either way), when two researchers visited participating dyads at home to collect outcome data; the participant and carer were interviewed separately (see table 1). During the covid-19 lockdown, this follow-up was undertaken remotely via telephone or video calls.

**Table 1:** Data collection time points

Scale or measure	Collection points			Discontinued from Mar 2020	Not collected remotely	Data provider
	Baseline	6 months	12 months			
Disability assessment for dementia (DAD)	Yes	–	Yes	–	–	Carer
Nottingham extended activities of daily living (NEADL)	Yes	–	Yes	–	–	Patient
Personal characteristics	Yes	–	–	–	–	Carer
Medical history	Yes	–	–	–	–	Carer
Medications	Yes	–	–	–	–	Carer
Montreal cognitive assessment (MoCA)	Yes	–	Yes	–	Yes	Patient
Verbal fluency	Yes	–	Yes	–	Yes	Patient
Cambridge neuropsychological test automated battery (CANTAB)	Yes	–	Yes	Yes	Yes	Patient
Apathy evaluation scale	Yes	–	Yes	–	–	Carer
Berg balance scale	Yes	–	Yes	–	Yes	Patient
Hand grip strength	Yes	–	Yes	–	Yes	Patient
Timed up and go (TUG) test and dual task TUG	Yes	–	Yes	–	Yes	Patient
SHARE (Survey of Health, Ageing, and Retirement in Europe) frailty instrument	Yes	–	Yes	–	Yes	Patient
Longitudinal Study of Ageing Amsterdam Physical Activity Questionnaire (LAPAQ)	Yes	–	Yes	–	–	Carer
Step count over 7 days (accelerometer)	Yes	–	Yes	Yes	Yes	Patient
Euroqol EQ-5D-3L self-completed quality of life	Yes	–	Yes	–	–	Patient
Euroqol EQ-5D-5L proxy completed quality of life	Yes	Yes	Yes	–	–	Carer
Dementia quality of life scale (DEMQOL)	Yes	–	Yes	–	–	Patient
Dementia utility weighted items (DEMQOL-U)	Yes	Yes	–	–	–	Carer
Dementia quality of life scale (DEMQOL-proxy)	Yes	–	Yes	–	–	Carer
Hospital anxiety and depression scale (HADS)	Yes	–	Yes	–	–	Patient
Falls efficacy scale–international (FES-I)	Yes	–	Yes	–	–	Patient
Carer strain index	Yes	–	Yes	–	–	Carer
Carer quality of life EQ-5D-5L	Yes	–	Yes	–	–	Carer
Client service receipt inventory	Yes	Yes	Yes	–	–	Carer

Calendars were collected monthly from randomisation to 15 months.

Falls, PrAISED activities done independently, service use, and hospital admissions were ascertained using monthly self-completed calendars between months 0 and 15, with researchers not involved in delivery of the intervention providing telephone prompts and support when necessary. Two clinicians adjudicated injurious falls based on details provided on calendars.

### Study outcomes

At six months, information was collected on quality of life (EQ-5D-3L, DEMQOL-U) and service use (short client service receipt inventory).

The primary outcome was carer (informant) rated disability in activities of daily living after 12 months, measured using the disability assessment for dementia scale. Secondary outcomes at 12 months were scores on the self-reported Nottingham extended activities of daily living scale; falls, rate of falling, and injurious falls; cognition (Montreal cognitive assessment scale, verbal fluency, apathy evaluation scale, and Cambridge neuropsychological test automated battery); quality of life (DEMQOL, EQ-5D); activity (Longitudinal Aging Study of Amsterdam Physical Activity Questionnaire, accelerometers); frailty (SHARE frailty instrument); Berg balance scale, functional mobility (single and dual task timed up and go test), and hand grip strength; fear of falling (short falls efficacy scale-international); mood (hospital anxiety and depression scale); and carer strain (caregiver strain index) and carer quality of life (see table 1).

### Harm and adverse events

Adverse events were classified as serious (death, life threatening events, hospital admission, substantial incapacity) or potentially related to the intervention or to PrAISED exercises undertaken independently. Adverse events were ascertained by participants or their carer reporting them to the study or intervention delivery teams or through the monthly calendars. The intervention group had more exposure to study therapists, and consequently more opportunity to report adverse events, resulting in an ascertainment (information) bias. To compare the safety of the intervention we considered deaths, hospital admissions, and falls to be core adverse events. To investigate the possibility of an early falls hazard associated with increased activity, we analysed falls in the first three months separately.

### Impact of the covid-19 pandemic

Following the UK Government’s guidance on strict social distancing and remaining at home during the covid-19 pandemic, all non-essential face-to-face contact ceased on 17 March 2020. At this time, 64 participants had completed the study, 187 were still actively participating, and 27 had been recruited but not started therapy. A series of mitigating measures was undertaken, and PrAISED therapists received guidance (supplementary appendix 2). A protocol amendment to adapt trial procedures was approved, including delivery of the intervention via telephone or videocall.^71^ As follow-up assessments were conducted remotely, we were unable to complete measures requiring physical contact. We removed some outcome measures to reduce burden on participants (see table 1). The final assessment was brought forward for participants within six weeks of the end of their programme. An additional interim outcome data collection point was introduced for all other remaining participants, in case no further trial activity became possible. Recruitment and in-person therapy and data collection restarted after 1 September 2020, if participants were willing, using personal protective equipment and excluding assessments that required close personal contact or sharing of equipment (including Cambridge neuropsychological test automated battery cognitive measures and accelerometers). Some remote assessment continued after this time on participant request.

### Sample size

An initial calculation based on variables from published literature suggested that a sample size of 368 participants (184 in each group), with a 23% attrition rate, had 80% power to detect changes in disability outcome (disability assessment for dementia scale), with an effect size of 0.5 (11 points on a baseline of 70 (standard deviation 22) points).^19^ ^72^ A minimum clinically important difference has not been defined for the disability assessment for dementia scale, but a natural history study in Alzheimer’s disease suggested the loss of about 1 point each month over 12 months.^73^ Following the feasibility study, a recalculation suggested that a sample size of 248 was sufficient. The original sample size was maintained with the agreement of the data monitoring and steering committees in the light of uncertainties in estimates. In the event, with the covid-19 pandemic, this proved prescient. Recalculation of sample sizes in July 2020, before restarting the trial, under a range of feasible impacts on intervention effect size, primary outcome standard deviation, and withdrawal rates, suggested that the sample size of 368 had adequate power to answer the research question.

### Randomisation

Randomisation was performed after baseline assessment and obtaining consent. A secure internet based system using a dynamic, adaptive allocation (minimisation) algorithm, accessed by a secure web portal held at the North Wales Organisation for Randomised Trials in Health Clinical Trials Unit, Bangor University, was used to randomise individuals, 1:1, stratified by site, presence of a co-resident, and history of previous falls. The adaptive allocation algorithm used a dynamic method of calculating allocation probabilities—that is, the probability of allocation to each group was not fixed (eg, 0.5) but was recalculated for every participant on the basis of participants already allocated. This method protects against subversion while ensuring that the trial maintains good balance to the allocation ratio of 1:1, both within each stratification variable and across the trial.^74^ A statistician independent of the analysis and research teams maintained the randomisation system. Allocation was emailed to the intervention delivery teams, who arranged the first clinical assessment.

### Blinding

Blinding of participants and therapists was not possible owing to the nature of the intervention. Researchers who collected outcome data were not blinded as the feasibility study showed that participants almost always inadvertently unblinded the researcher.^22^ Analysis was, however, done blinded.

### Statistical analysis

For the primary outcome of difference in disability assessment for dementia score between groups, an analysis of covariance was conducted using stratification variables (site, co-resident carer, and history of falls) and baseline disability assessment for dementia score as covariates. For secondary outcome measures, analyses of covariances were conducted, using the stratification and respective baseline measures as covariates. All analyses were done on an intention-to-treat basis, although data were only available for people who completed the outcome assessment. For the main analysis, multiple imputation using chained equations was used when <40% of the data were missing and data were not missing completely at random. Number of imputations was based on proportion of missing data for the outcome. The imputation models included the randomisation stratification variables, participant’s baseline characteristics, and scores at baseline. Imputation was done overall rather than separately by randomised group. Results for the imputed dataset were pooled using Rubin’s rule. Effect sizes were standardised as Cohen’s d.^75^ Adjusted mean differences, effect size estimates, 95% confidence intervals, and P values were reported for all analyses.

Multiple sensitivity analyses were conducted: including complete cases; including those who had completed the trial before the covid-19 pandemic; using the interim data collected in the early weeks of the pandemic; correcting for survivor bias, by assigning a disability assessment for dementia score of zero to participants who died; excluding those who terminated the intervention early owing to the pandemic; and excluding three participants who had Montreal cognitive assessment scores at baseline above the upper limit.

Falls were analysed as the proportions of participants who fell, the incidence rate ratio using a negative binomial regression, and time to first fall using a Cox proportional hazards regression. We anticipated that any impact of the PrAISED intervention on falls would not be immediate, so our predefined efficacy outcome was rate of falling between months 4 and 15. Only participants with a complete series of calendar returns were included.

The statistical analysis plan is available, including further details of imputation methods.^76^

### Process evaluation

We undertook a process evaluation in accordance with Medical Research Council guidelines.^77^ ^78^ ^79^ ^80^ Reach, dose, fidelity, and adaptations of training and intervention delivery were investigated (supplementary appendix 2). We recorded details of each session delivered, exercise undertaken independently via monthly calendars, and fidelity of delivery from analysis of a sample of 14 video recorded sessions, in which evidence of the 14 core principles of the PrAISED intervention was sought.^52^ Qualitative interviews were conducted with a sample of participants with dementia, carers, and therapists to investigate how the intervention was received as well as barriers and facilitators to participation.

An independent steering committee and a data monitoring committee monitored the trial.^81^

### Patient and public involvement

Patient and public involvement was integrated into every stage of the research cycle, with the aim that the intervention had relevance and the research processes were acceptable to people with mild dementia and their carers. One of our co-investigators was a carer. Patient and public involvement representatives were members of the programme management group and the trial steering committee. They worked in collaboration with the research team to develop the funding application and intervention, co-designed patient facing materials, participated in research interviews,^82^ and helped interpret results. Intervention burden was assessed qualitatively in a process evaluation.^80^

## Results

**Fig 1:**
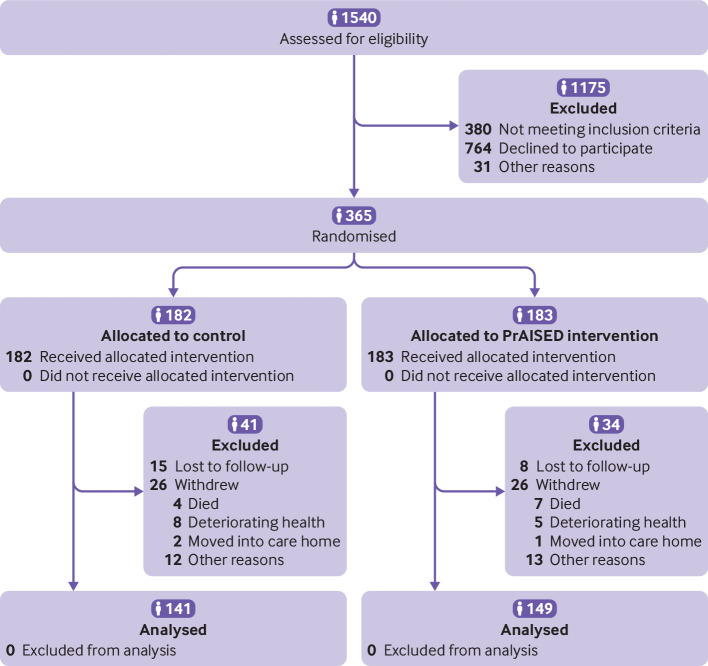
Flow of participants through study

From 8 October 2018 to 23 June 2022, 1540 potential patient participants were screened, of whom 319 were ineligible and 746 did not wish to take part. Of 475 screened, 110 were not randomised: 61 were ineligible, 18 withdrew, and 31 were lost (fig 1). Overall, 365 patient and 365 carer participants were randomised (84 (23%) in Bath, 79 (22%) in Derby, 60 (16%) in Lincoln, 23 (6%) in Oxford, and 119 (33%) in Nottingham). Participants were recruited from memory clinics (288 (79%)), GP registers (40 (11%)), post-diagnostic support services (15 (4%)), and the Join Dementia Research register (22 (6%)). Three protocol deviations involved failure to adjust baseline Montreal cognitive assessment scores for duration of education.

Seventy five participants (21%) did not complete the 12 month follow-up: 52 (14%) withdrew and 23 (6%) were lost. The number of withdrawals did not differ between groups (26 *v* 26, Fisher’s exact test P=0.9). A blinded panel assessed available details for participants who withdrew to determine whether they had meaningful health related outcomes—that is, had died, been admitted to a care home, or withdrew because of deteriorating health. Overall, 27 withdrawals were meaningful, with no difference between groups (14 *v* 13, P=1.0).

### Baseline data

Baseline characteristics were similar between groups, including those potential participants who withdrew (table 2). The median age of patient participants was 80 years (range 65-95), 210 (58%) were men, 358 (98%) were of white ethnicity, 247 (68%) were married, and 276 (76%) lived with a carer. 113 (31%) had completed a college or university degree. 70 (19%) had mild cognitive impairment, 142 (39%) had Alzheimer’s dementia, 71 (19%) had vascular dementia, 59 (16%) had mixed dementia, and 21 (7%) were classed as having other or unknown conditions. The median age of the carer participants was 70 years (range 20-94), 236 (65%) were the spouses of the patient participant, and 265 (73%) were female carers. 124 (34%) of carers had a limiting long term health condition.

**Table 2:** Baseline variables by allocation group. Values are number (percentage) unless stated otherwise

Characteristics	Control (n=182)	Intervention (n=183)	Withdrew (n=52)
**Patient participants**			
Median (IQR) age (years)	81 (75-84)	80 (75-85)	80 (77-85)
Female sex	73 (40)	82 (45)	23 (44)
White ethnicity	179 (98)	179 (98)	52 (100)
Married	123 (68)	124 (68)	34 (66)
Lives alone	46 (25)	43 (24)	17 (33)
Mean (SD) Montreal cognitive assessment score*	19.8 (3.1)	20.0 (3.2)	19.9 (3.2)
Degree or higher	54 (30)	59 (32)	12 (23)
Diagnosis of dementia	142 (78)	151 (83)	44 (85)
Mean (SD) comorbidity count†	4.0 (2.0)	3.9 (1.8)	3.9 (1.9)
Mean (SD No of drugs	6.1 (3.5)	6.1 (3.2)	6.1 (3.4)
Previous fall	95 (52)	93 (53)	26 (50)
**Carer participants**			
Husband, wife, or partner	119 (65)	117 (64)	32 (62)
Son or daughter	50 (28)	55 (30)	16 (31)
Other	13 (7)	11 (6)	4 (8)
Female carer	134 (74)	131 (72)	38 (73)
Co-resident carer	136 (75)	140 (76)	38 (73)
Carer with long term medical condition	63 (35)	61 (33)	20 (39)
Median (IQR) carer age (years)	70 (58-77)	70 (58-78)	69 (58-78)

IQR=interquartile range; SD=standard deviation.

* Maximum score 30.

† Maximum count 23.

### Adherence and fidelity

The intervention was delivered largely as intended, and participants engaged well despite the disruption caused by the covid-19 pandemic. Participants in the intervention group took part in a median of 31 therapy sessions (interquartile range 22-40). The mean length of sessions was 71 minutes (standard deviation (SD) 30; range 5-220). Two thirds of sessions were delivered face-to-face (1357 (68%)). Fidelity of therapy delivery was 70% against PrAISED core principles rated from video recordings. In total, 4040/4863 (83%) of the expected calendars were returned. The intervention group participants reported completing a mean of 482 minutes of PrAISED exercise per month (SD 705; range 0-5310; 121 minutes/week). Of control group participants, 95 (54%) had one therapy session, 48 (27%) had two sessions, 29 (16%) had three sessions, 4 (2%) had four sessions, and one had five sessions.

### Outcomes

The primary activities of daily living outcome (disability assessment for dementia) did not differ between intervention and control groups: adjusted mean difference −1.3, 95% confidence interval −5.2 to 2.6; Cohen’s d effect size −0.06, 95% confidence interval −0.26 to 0.15, P=0.51), or on most secondary outcome measurements, including balance, functional mobility, physical activity, and quality of life (table 3 and table 4). Upper 95% confidence intervals excluded small to moderate beneficial treatment effects. Statistically significant small differences were in favour of the control group on the dual task timed up and go test (d=−0.48, −0.12 to −0.83, P=0.01) and self-reported DEMQOL (d=−0.26, −0.47 to −0.06, P=0.01), but not DEMQOL proxy or Euroqol EQ-5D quality of life measures.

**Table 3:** Unadjusted scores on outcome measures according to randomisation group

Study group by measure	No of participants	Mean (SD) score		Difference
		Baseline	Follow-up	
Activities of daily living (score ≤100)*:				
Control	125	77.8 (20.8)	66.4 (24.5)	−11.4
Intervention	133	77.6 (20.1)	64.2 (25.7)	−13.4
Activities of daily living (NEADL) (score ≤22)*:				
Control	124	16.8 (3.9)	14.1 (4.8)	−2.7
Intervention	129	16.2 (4.2)	13.9 (4.3)	−2.2
Physical activity (LAPAQ)*:				
Control	118	1483 (1608)	1293 (1430)	−189
Intervention	130	1395 (1230)	1037 (1224)	−358
Accelerometer (No of steps in 7 days)*:				
Control	22	21 412 (20 112)	21 694 (17 308)	282
Intervention	21	24 410 (21 081)	20 584 (15 226)	−3826
Berg balance scale (score ≤56)*:				
Control	58	50.3 (5.5)	46.7 (10.6)	−3.6
Intervention	66	46.8 (9.6)	46.3 (9.2)	−0.5
Timed up and go (sec)†:				
Control	69	13.9 (6.7)	14.0 (7.0)	0.1
Intervention	69	13.7 (4.5)	16.6 (12.6)	3.0
Dual task timed up and go (sec)†:				
Control	62	18.4 (8.3)	20.8 (9.9)	2.3
Intervention	64	19.7 (11.7)	28.1 (20.0)	8.4
DEMQOL self-reported (score ≤112)* (MCID 6):				
Control	136	90.9 (11.4)	88.9 (14.9)	−2.0
Intervention	140	89.2 (12.9)	83.7 (15.2)	−5.5
DEMQOL proxy (score ≤124)* (MCID 6):				
Control	135	95.6 (12.9)	90.7 (15.1)	−4.9
Intervention	145	92.1 (13.3)	90.6 (13.3)	−1.5
DEMQOL-U (6 months)*:				
Control	149	0.69 (0.1)	0.72 (0.13)	0.03
Intervention	141	0.72 (0.1)	0.72 (0.13)	0
Self-reported quality of life EQ-5D-3L (score ≤1.0)*:				
Control	135	0.82 (0.18)	0.75 (0.25)	−0.07
Intervention	138	0.81 (0.18)	0.75 (0.24)	−0.06
Proxy quality of life EQ-5D-5L (score ≤1.0)*:				
Control	130	0.80 (0.18)	0.71 (0.23)	−0.09
Intervention	143	0.79 (0.17)	0.73 (0.19)	−0.07
Montreal cognitive assessment (score ≤30)*:				
Control	77	20.0 (3.2)	17.5 (4.6)	−2.5
Intervention	75	20.1 (3.5)	17.3 (5.2)	−2.8
Verbal fluency/words*:				
Control	79	12.3 (4.7)	10.8 (4.8)	−1.5
Intervention	76	12.0 (4.6)	10.0 (4.4)	−1.9
Apathy evaluation scale (score ≤72)†:				
Control	121	40.3 (12.0)	44.6 (12.0)	4.3
Intervention	134	42.4 (12.4)	46.3 (13.0)	3.9
Falls efficacy scale-international (score ≤28)†				
Control	134	10.3 (4.1)	10.9 (4.8)	0.5
Intervention	138	10.4 (3.9)	11.0 (4.5)	0.6
HADS anxiety (score ≤21)†:				
Control	133	3.7 (2.9)	4.2 (3.6)	0.5
Intervention	132	4.3 (3.0)	5.0 (3.3)	0.7
HADS depression (score ≤21)†:				
Control	132	3.9 (2.6)	4.8 (3.7)	0.9
Intervention	132	4.9 (2.7)	5.3 (3.0)	0.4
SHARE frailty index†:				
Control	71	1.6 (1.6)	1.7 (1.8)	0.1
Intervention	72	1.7 (1.8)	1.7 (1.8)	0
Hand grip strength right hand (kg)*:				
Control	78	24.9 (10.8)	23.6 (9.6)	−1.3
Intervention	76	22.4 (8.3)	20.9 (7.5)	−1.5
Carer strain index (score ≤13)†:				
Control	125	4.3 (3.3)	4.7 (3.5)	0.4
Intervention	134	4.7 (3.3)	4.8 (3.5)	0.2
Carer EQ-5D-5L index (score ≤1.0)*:				
Control	132	0.88 (0.17)	0.86 (0.17)	−0.02
Intervention	140	0.85 (0.18)	0.85 (0.16)	−0.01

DAD=disability assessment for dementia; NEADL=Nottingham extended activities of daily living; MCID=minimum clinically important difference; DEMQOL=dementia quality of life; LAPAQ=Longitudinal Ageing Study Amsterdam Physical Activity Questionnaire; EQ-5D-3L=EuroQol five dimensions questionnaire-three levels; EQ-5D-5L=EuroQol five dimensions questionnaire–five levels; HADS=hospital anxiety and depression scale; SHARE=Survey of Health Ageing and Retirement in Europe.

* Higher score superior.

† Higher score inferior.

**Table 4:** Analysis of covariance and standardised effect size estimates for intervention group (with missing data imputed)

Measures	No of participants	Adjusted mean difference (95% CI)*	Cohen’s d effect size (95% CI)†	P value
Disability assessment for dementia score	365	−1.3 (−5.2 to 2.6)	−0.06 (−0.26 to 0.15)	0.51
NEADL score	256	0.2 (−0.7 to 1.1)	0.05 (−0.20 to 0.29)	0.71
LAPAQ physical activity score	365	−167 (−445 to 112)	−0.14 (−0.35 to 0.06)	0.25
Accelerometer (No of steps in 7 days)	43	−4030 (−11 028 to 2969)	−0.37 (−0.98 to 0.23)	0.25
Berg balance scale	145	1.8 (−0.7 to 4.2)	0.15 (−0.08 to 0.57)	0.15
Timed up and go	138	−2.7 (−5.9 to 0.5)	−0.29 (−0.62 to 0.05)	0.10
Dual task timed up and go	126	−7.3 (1.8 to 12.8)	−0.48 (0.12 to 0.83)	0.01
DEMQOL self-reported	365	−3.8 (−6.8 to −0.8)	−0.26 (−0.47 to −0.06)	0.01
DEMQOL proxy	365	2.4 (−0.3 to 5.1)	0.18 (−0.03 to 0.38)	0.08
DEMQOL-U (6 months)	365	0.01 (−0.01 to 0.04)	0.11 (−0.1 to 0.3)	0.29
EQ-5D-3L index self-reported	365	0.02 (−0.04 to 0.07)	0.08 (−0.12 to 0.29)	0.51
EQ-5D-5L index proxy	365	0.03 (−0.01 to 0.07)	0.15 (−0.05 to 0.36)	0.16
Montreal cognitive assessment	155	−0.4 (−1.5 to 0.8)	−0.11 (−0.42 to 0.21)	0.52
Verbal fluency-correct words	155	−0.5 (−1.6 to 0.5)	−0.16 (−0.48 to 0.15)	0.32
Apathy evaluation scale	365	−0.6 (−2.7 to 1.4)	−0.07 (−0.27 to 0.14)	0.54
Falls efficacy scale-international, short	365	0.2 (−0.7 to 1.0)	0.05 (−0.15 to 0.26)	0.64
HADS anxiety	275	0.4 (−0.3 to 1.1)	0.15 (−0.09 to 0.38)	0.23
HADS depression	275	−0.1 (−0.8 to 0.6)	−0.03 (−0.27 to 0.20)	0.78
SHARE frailty instrument	149	−0.05 (−0.52 to 0.42)	−0.04 (−0.36 to 0.29)	0.56
Hand grip strength (kg)	154	−0.9 (−2.9 to 1.1)	−0.15 (−0.47 to 0.16)	0.36
Carer strain index	365	−0.01 (−0.63 to 0.62)	−0.04 (−0.25 to 0.16)	0.69
Carer EQ-5D-5L index	365	0.01 (−0.01 to 0.04)	0.09 (−0.12 to 0.29)	0.37

CI=confidence interval; NEADL=Nottingham extended activities of daily living; DEMQOL=dementia quality of life; LAPAQ=Longitudinal Ageing Study Amsterdam Physical Activity Questionnaire; EQ-5D-3L=EuroQol five dimensions questionnaire-three levels; EQ-5D-5L=EuroQol five dimensions questionnaire–five levels; HADS=hospital anxiety and depression scale; SHARE=Survey of Health Ageing and Retirement in Europe.

Imputation was not appropriate for variables showing fewer than 365 cases.

* Adjusted for age, sex, site, falls history, co-resident carer, and baseline score.

† 0-0.2=no effect; 0.2-0.5=small; 0.5-0.8=moderate; >0.8=large. Positive values show an effect in favour of intervention group.

The analyses of cognitive measures using the Cambridge neuropsychological test automated battery were underpowered but suggested statistically significant benefits for the PrAISED intervention, with a moderate effect size, on tests of multi-tasking (an executive function test assessing participants’ ability to manage conflicting information) and spatial span (a test of visuo-spatial working memory capacity; see supplementary appendix 4 and tables A1 and A2).

The sensitivity analyses, including complete cases, showed no differences in results (fully reported in supplementary appendix 5). The disability assessment for dementia score was higher when follow-up was face-to-face compared with data collected remotely (mean 72 *v* 63), but this was the same for both intervention and control groups. Overall, 10 (4.4%) of participants reported a confirmed covid-19 infection. In total, 185 (82%) engaged in social distancing for a median 116 days (interquartile range 37-210) and 107 (47%) reported self-isolated for a median 71 (22-139) days. Results did not differ between those reporting and those not reporting a covid-19 infection.

### Falls

Of 796 falls in total, 375 occurred in the intervention group and 421 in the control group. At least one fall was experienced by 60% of participants in the intervention group during the trial compared with 57% in the control group (odds ratio 1.1, 95% confidence interval 0.71 to 1.82, P=0.6). Seventy three falls were injurious: 38 in the intervention group and 35 in the control group. At least one injurious fall was reported by 15% of participants in the intervention group and 16% in the control group (odds ratio 0.91, 0.51 to 1.62, P=0.8).

The falls efficacy outcome concerned falls in months 4-15. For participants who completed all the study calendars for these months (n=128), 279 falls were recorded (79 intervention group, 200 control group). At least one fall was experienced by 59% of participants in the intervention group compared with 55% of participants in the control group (odds ratio 1.17, 0.57 to 2.40, P=0.72). The incidence rate for falls was 1.49 per person year in the intervention group compared with 2.47 per person year in the control group: incidence rate ratio 0.78, 95% confidence interval 0.46 to 1.31, P=0.33, adjusted for site, co-resident carer, and history of falls. A survival analysis showed median time to the first fall was 13 months in the intervention group and 12 months in the control group (adjusted hazard rate ratio 0.85, 95% confidence interval 0.50 to 1.43, P=0.54).

### Harms (adverse events)

One hundred and sixty seven adverse events were recorded (59 in the control group, 108 in the intervention group), involving 68 participants: 27 (15%) in the control group and 61 (33%) in the intervention group. Ninety one serious adverse events occurred (29 in the control group, 62 in the intervention group), involving 60 participants: 22 (12%) in the control group and 38 (21%) in the intervention group. None of the adverse events was related to treatment. Eleven deaths occurred: four (2.2%) in the control group and seven (3.8%) in the intervention group (odds ratio 2.25, 0.70 to 8.72, P=0.26). Seven new care home placements took place: two (1.1%) from control and five (2.7%) from intervention group (odds ratio 2.42, 0.49 to 18.97, P=0.45). Seventy five patient participants were admitted to hospital (27 in the control group, 48 in the intervention group), involving 53 participants: 22 (12%) in the control group and 31 (17%) in the intervention group (odds ratio 1.48, 0.82 to 2.70, P=0.24).

For participants who completed calendars for the first three months, 228 falls were recorded (132 in the control group, 96 in the intervention group). At least one fall was experienced by 32% of participants in the intervention group compared with 31% of participants in the control group (odds ratio 1.07, 0.64 to 1.79, P=0.90).

## Discussion

For people with mild dementia or mild cognitive impairment, the PrAISED intervention did not improve activities of daily living, physical activity, quality of life, or any other health status outcome, including balance and functional mobility in the 12 month period after randomisation. There may have been a small reduction in rate of falling (22% relative risk reduction, statistically uncertain), and improvement in some specific cognitive domains, in underpowered analyses, but these did not translate into functional gains. Delivery of the intervention was disrupted by restrictions due to the covid-19 pandemic.

### Strengths and limitations of this study

This was a high quality multicentred randomised controlled trial. We followed Medical Research Council guidance to develop and evaluate the intervention.^83^ Before starting the trial we established the feasibility and acceptability of intervention delivery and trial processes.^22^ A process evaluation established reasonable participant adherence and fidelity of intervention delivery. Our attrition and missing data rates were good. In qualitative interviews, undertaken as part of the process evaluation, the trial intervention was overwhelmingly well received by participants, carers, and provider staff.^80^

The intervention was systematically designed and refined over several years, including during the feasibly trial.^21^ ^22^ ^54^ It was intended to be practical and relevant to participants. The intervention comprised predominantly resistance (strength and balance) exercises in a home setting, linking to daily activities, explicitly addressing risk of falls and other safety concerns, and encouraging outdoor mobility. The intervention was individualised (tailored, personalised). Exercise was not a standard prescription but was seen as subserving activities that participants needed or wanted to do. Close attention was paid to motivation, the learning needs of people with dementia, and contextual factors, especially involvement of family or other carers. The intervention was delivered by trained and experienced physiotherapists and occupational therapists, who made assessments and plans and supervised trained rehabilitation support workers.

The intervention was about as intensive as could be credibly delivered by a public health service. The funder, the National Institute for Health and Care Research on behalf of the UK NHS, was concerned that the intervention was unfeasibly intensive and requested the inclusion of a briefer and less expensive intervention in the feasibility study. The feasibility study, however, demonstrated the need for prolonged supervision.^22^ In our main trial, we emphasised tailoring of supervision to individual needs. Although a median of 31 therapy sessions over a year might have been insufficiently intensive to change outcomes (compared with, for example, 104 sessions in the Finnish Alzheimer Disease Exercise Trial (FINALEX)^19^), it was probably the maximum plausible dose in relation to NHS services and costs of delivery. We used tapering of the intervention, with twice weekly visits in the first three months, reducing to monthly visits in the last three months to encourage independent undertaking of exercise. In the event, without direct supervision this could have reduced adherence.

The patient population lacked diversity, being disproportionately well educated, white men. The study enrolled people willing to agree to take part in research and perform prolonged exercise who may already have had healthy lifestyles and therefore been the least likely to benefit. Motivation to take part in trials is unlikely to be independent of motivation to exercise.

The trial was disrupted by the covid-19 pandemic and the associated lockdown and social distancing. Clinical delivery teams were quick to move to remote delivery of the intervention and in so doing demonstrated great flexibility and innovation,^71^ ^84^ but this type of delivery did not work for many participants, such as those with sensory impairments, those who lacked information technology hardware or internet connections, and those with no carer to help with telephone calls or videoconferencing. In this situation, progression of exercises was impossible to do safely, and access to community facilities diminished or ceased. Some follow-up interviews were conducted remotely, which might have affected data quality. Remote follow-up prevented us from collecting some secondary outcome measures. Subgroup analysis on participants followed-up before the covid-19 pandemic did not suggest different results, however. Equally, it could be argued that the pandemic was challenging for all older people, and our intervention could have mitigated this and shown exaggerated benefits.^85^ ^86^

Blinding of participants was not possible, as is usual in rehabilitation trials, although both groups had an active intervention. In the feasibility study, we tried blinding the researchers who collected outcome data, but this proved impossible to maintain. Participants’ expectations of the outcome of intervention or usual care were not measured but were explored in qualitative interviews.^80^ Social desirability or expectation biases might lead intervention participants to overstate, and control participants to understate, their functioning, thereby exaggerating the measured treatment effect. This risk of bias is unlikely to alter our conclusion of no or negligible effects of the intervention. Moreover, disability assessment for dementia is standardised, researchers were trained in how to administer the intervention, and guidelines for interpretation were issued. We planned to objectively measure physical activities undertaken by participants in their own time using accelerometers but had to abandon this owing to the pandemic.^87^ We had no direct measure of participant independence, nor participant or carer satisfaction with the programme.

### Research in context

Numerous reviews of non-drug interventions in dementia have been published. Evidence that exercise and physical activity can improve activities of daily living for people with dementia is inconclusive. A Cochrane review found no high quality evidence.^16^ A further review concluded that exercise and physical activity reduced disability and falls but that the quality of evidence was low.^88^ A recent meta-analysis found no effect of exercise on activities of daily living.^89^ Two reviews considered a range of interventions designed to maintain functional activity in dementia. Both identified heterogeneity between studies, mixed evidence of effectiveness, generally low quality of evidence, but a greater effect when interventions were tailored to participants’ interests and abilities and delivered by registered therapists.^90^ ^91^ The evidence for moderate to high intensity exercise preventing falls in cognitively intact older people is strong.^92^

Some adequately powered and high quality individual trials have been performed. One trial of prolonged (12 months) and intensive (one hour twice a week), physiotherapist supervised, home exercise for people with Alzheimer’s disease found a substantial reduction in rate of loss of activities of daily living abilities and halved the rate of falling.^19^ Two trials of exercise interventions for people with sarcopenia and frailty who were not cognitively impaired showed small but significant improvements in the incidence of mobility disability (20% risk reduction) and frailty markers with moderate intensity programmes.^93^ ^94^ ^95^ Two trials that investigated moderate to high intensity supervised group exercise over four months for people with mild to moderate dementia showed no improvement in activities of daily living after six months.^96^ ^97^ Functionally oriented occupational therapy improved abilities and activity,^60^ but these findings were not replicated in two subsequent trials.^98^ ^99^ The results of the Journeying Through Dementia trial of a bespoke, moderate intensity, occupational therapy intervention were negative.^100^ A trial of cognitive rehabilitation in mild to moderate dementia, focusing on functional activity, showed that more goals were met in the intervention group compared with a control group, but there was no impact on health status measures such as activities of daily living.^101^

### Interpretation of the findings

Dementia is a progressive condition with no cure. In recent years, interest in preventing dementia has been increasing.^14^ Secular trends in incidence suggest that dementia risk is not immutable,^6^ but good evidence for the effectiveness of interventions to reduce dementia risk is lacking. Protective factors such as physical activity are likely to act over decades rather than months. Secondary prevention (of progression once dementia is diagnosed) through lifestyle interventions seems to be ineffective. A reduction in rate of falling remains possible and may be valuable, but it did not impact on preservation of activities of daily living or quality of life. The point estimate of falls risk reduction in our study was in line with estimates from meta-analyses.^92^

We found improvements in some aspects of cognitive function, but these were small, and the analyses were underpowered. Any benefits did not contribute to better functional ability. Cognitive stimulation was not used in our intervention, apart from dual task training, which can be considered to be training in divided attention. The occupational therapy approach was a form of cognitive rehabilitation.

Current health policy emphasises living well after a dementia diagnosis, through a combination of healthcare, psychosocial and societal changes, and adaptation of services to meet the particular needs of people with dementia.^10^ We and others have shown that maintaining abilities is not likely to be possible. This does not mean that intervention may not have benefit in the psychosocial domain, including affirming personhood, inclusion, occupation, relationships, or carer support. Aspects such as social engagement, concern, hope, achievement of goals, information giving (on a range of dementia related topics), and therapeutic relationship building seem to have been greatly valued.^80^ In palliative care and mental health, therapeutic relationships are valued in their own right; in our study the exercise may have been a means to an end.

The absence of measurable health gain makes it hard to argue for routine provision of this intervention. Our observations could, however, inform the development of future models of support. A widespread perception exists of a service gap for people after a dementia diagnosis and their carers. The healthcare background and knowledge and expertise of the therapists seems to have been relevant to delivering holistic and supportive intervention. What this means in terms of measurement and evaluation is yet to be defined, but our current paradigm may be missing something. Others have commented on the unsuitability of available outcome measures and the limitations of randomised controlled trials in evaluating interventions in this population.^99^ ^100^ A social return on investment analysis of our feasibility trial, a health economic methodology which attempts to identify, quantify, and monetise a wide range of health, personal, and social benefits from a public policy perspective, was strongly positive.^102^

Some specific aspects of our trial may explain negative results. The disability assessment for dementia scale is recommended as the most appropriate activities of daily living outcome for dementia trials, but it can be difficult to complete. The scale distinguishes between initiation and performance of activities, and privileges activities undertaken without prompting. Although reasonable on a normative basis, this privileging of unsupported activity may not adequately ascertain supported performance rather than independence. We undertook training in dual task activities, as impairment in these is a risk factor for falling, they are trainable, and improved abilities can carry over between activities.^56^ ^57^ ^58^ Our main index of ability in dual tasking was the dual task timed up and go test, the results of which deteriorated in the intervention group. The test involved getting up, walking 3 m, turning, and sitting down again while counting backwards. The instructions were cognitively demanding. Researchers reported that participants who had received active therapy sometimes misunderstood the task, such as trying to walk backwards, and may have confused the test with therapeutic tasks they had practised during the intervention. Similarly, the DEMQOL quality of life scale asks if participants are worried about things related to their dementia. The therapy programme may have increased participants’ awareness of their inabilities. That said, the most likely explanation for the difference in DEMQOL scores was chance. The difference was small and was not supported by the other measures of quality of life, some of which changed in the opposite direction. We observed a small but consistent excess of harms associated with the intervention (deaths, care home placements, hospital admissions, adverse events). Interpreting adverse events was difficult owing to information bias, because the intervention group had far more opportunity to report them. All adverse events were scrutinised and none appeared to be related to the intervention. We have been unable to determine any pattern, or reason for this difference, and think that it is also most likely due to chance.

We can speculate whether control group participants who receive a diagnosis of dementia and who are sufficiently able to volunteer for a research study, successfully adapt, drawing upon their existing resources and striking a balance that works for them in terms of activity and wellbeing. The objective of the trial was to introduce an intervention that did something different. Frequent involvement of healthcare professionals could, however, disrupt normal adaption, draw attention to ill health rather than wellbeing, encourage people to take greater risks than they would usually have done, and prompt greater involvement of healthcare services. The intervention which the control group received was more than would be delivered to people with dementia in routine practice but was less than is established to reduce the risk of falls or improve executive function and involved substantially less professional contact than the intervention group received. The rationale was to improve retention in the control group by avoiding the resentful disengagement reported in some trials. The calendar data indicated that the intervention group exercised substantially more than the control group. Furthermore, in our three arm feasibility trial, an intervention comprising 13 visits over three months proved inadequate to enable participants to sustain activity and engagement, so it is unlikely that the few sessions of the control condition in this trial would do so.^22^

### Implications and future work

We add to accumulating evidence that interventions to delay cognitive or functional decline in early dementia or mild cognitive impairment are ineffective. So far, drug therapies, cognitive stimulation, exercise, and rehabilitation therapies have, at best, a small impact on functional activities and quality of life and do not appear to change the course of the disease. Recent data on lecanemab, the monoclonal antibody drug used in the treatment of Alzheimer’s disease, found small benefits in activities of daily living (2 points on a 90 point scale over 18 months).^104^

We need to think again about how we support people with dementia to live well. A more supportive approach to care may be required. Healthcare interventions should focus on solving practical problems and crises. Emphasis should be on helping individuals with dementia to live well despite their limitations; minimise intervention burden; maintain personhood, inclusion, and occupation; provide psychological and emotional support; and support family and other carers. Restoration of independence in activities may be unrealistic; adapted or supported functioning (compensatory approaches) may be more achievable.^103^ For example, individuals may be assisted to cook or shop so that that they remain active and included, rather than aiming for them to be able to do these tasks alone. Outcome measures that reflect these are needed. The value of therapeutic relationships may be underappreciated and may go beyond what might be expected from befriending, counselling, or social prescribing. Exercise and physical activities should be promoted for enjoyment, occupation, and inclusion and to enhance relationships.

**What is already known on this topic**

Dementia is associated with progressive loss of functional ability, including activities of daily living and mobility, and a high risk of falls
Exercise programmes and rehabilitation therapies may improve ability, or slow the rate of decline, but evidence from trials and systematic reviews is equivocal

**What this study adds**

An intensive dementia specific exercise and functional activity rehabilitation programme was well received by participants and therapists
The intervention had no effect on activities of daily living, physical activity, quality of life, falls, cognition, or any other health status outcome
Loss of ability in dementia is unlikely to change through exercise or functionally oriented rehabilitation therapy

## Data Availability

Data sharing might be possible for additional analyses by contacting the corresponding author.

## Web extra

Extra material supplied by authors

Supplementary information: statistical report

## Notes

Contributors: RH, TM, SG, VvdW, PL, HS, KP, VHM, KV, RdN, MG, ZH, RTE, JG, KP, and MO conceived and designed the study and obtained funding. VB, PL, TB, LH, AC, RT, JH, CDL, HS, LB, AL, MG, MD, TM, and RH were responsible for intervention development, training, and delivery support. CDL, VB, VvdW, JH, FK, SG, and RH searched the literature. RB, CB, JL, AL, ST, KJ, CD, CDL, AHS, and TW undertook recruitment and data collection. ZH, AB, and RH were responsible for the statistical design and analysis. CDL, VvdW, KP, MG, MD, VB, LH, and RH undertook the process evaluation. RTE, VE, and NH undertook the health economic design and evaluation. SG, VvdW, ROB, EA, MG, MD, KK, RV, TM, and RH were responsible for study management. RH, CDL, VvdW, SG, FK, PL, JG, KP, VB, LH, AC, EA, RdN, TW, HS, MO, MG, and MD interpreted the study. RH, SG, FK, and CDL drafted the manuscript. All authors approved the final paper. RH is the guarantor. The corresponding author attests that all listed authors meet authorship criteria and that no others meeting the criteria have been omitted.

The lead author affirms that the manuscript is an honest, accurate, and transparent account of the study being reported; that no important aspects of the study have been omitted; and that any discrepancies from the study as originally planned have been explained.

Dissemination to participants and related patient and public communities: a newsletter summarising the results was sent to participants.

Provenance and peer review: Not commissioned; externally peer reviewed.
